# Increased Tissue Expression of Lectin-Like Oxidized LDL Receptor-1 (LOX-1) Is Associated with Disease Severity in Chronic Rhinosinusitis with Nasal Polyps

**DOI:** 10.3390/diagnostics10040246

**Published:** 2020-04-23

**Authors:** Manabu Nishida, Sachio Takeno, Kohta Takemoto, Daisuke Takahara, Takao Hamamoto, Takashi Ishino, Tomohiro Kawasumi

**Affiliations:** Department of Otorhinolaryngology, Head and Neck Surgery, Graduate School of Biomedical Sciences, Hiroshima University, Kasumi 1-2-3, Minami-ku, Hiroshima 734-8551, Japan; nh1027@hiroshima-u.ac.jp (M.N.); d192677@hiroshima-u.ac.jp (K.T.); pujols@hiroshima-u.ac.jp (D.T.); takao0320@hiroshima-u.ac.jp (T.H.); tishino@hiroshima-u.ac.jp (T.I.); hu0401tk@hiroshima-u.ac.jp (T.K.)

**Keywords:** chronic rhinosinusitis with nasal polyps, CRSwNP, lectin-like oxidized LDL receptor-1, LOX-1, oxidative stress, reactive oxygen species, ROS, scavenger receptors, scavenger receptor class B type 1, SR-B1

## Abstract

Background: The oxidative stress, induced by both environmental and intrinsic stimuli, underlies the onset and persistency of chronic rhinosinusitis (CRS). Scavenger receptors (SRs) are a broad family of transmembrane receptors involved in a dysfunctional host–environment interaction through a reaction with reactive oxygen species (ROS) production. Objective: We hypothesized possible roles of two major SRs in CRS pathology that can translate to clinical phenotypes or histological subtypes: lectin-like oxidized low-density lipoproteins (LDL) receptor-1 (LOX-1) and scavenger receptor class B type 1 (SR-B1). Patients and Methods: We collected ethmoid sinus mucosa specimens and blood samples from patients with CRS with nasal polyps (CRSwNP; n = 31) or CRS without NP (CRSsNP; n = 13) and 19 control subjects. We performed an RT-PCR analysis, ELISA assay, and immunostaining to determine the expressions and distributions of LOX-1 and SR-B1. Results: The CRSwNP group showed a significant increase in LOX-1 mRNA expression compared to the control group. There was no significant difference in SR-B1 mRNA levels among the three groups. The LOX-1 mRNA levels were positively correlated with the sinus computed tomography (CT) scores. Sinus tissue, but not serum samples, showed elevated concentrations of LOX-1 protein in the CRSwNP group versus the control group. The LOX-1 protein distribution was localized in inflammatory cells and vascular endothelial cells. Conclusion: LOX-1 is a major receptor for oxidized low-density lipoprotein produced by oxidative stress. This is the first study to report alterations in LOX-1 expression and production triggered by persistent inflammatory processes in CRSwNP patients. Our findings reveal complex but important roles for SRs that may contribute to the onset of different CRS phenotypes.

## 1. Introduction

Oxidative stress underlies the onset and persistency of human airway inflammation. Exposure to various environmental agents induces oxidative stress and the production of reactive oxygen species (ROS) that have the capacity to cause tissue damage and accentuate airway inflammation [1,2,3]. Scavenger receptors (SRs), which were originally identified in human macrophages, recognize modified low-density lipoproteins (LDL) to develop foam cells [1]. SRs have structural diversity, with different groups categorized as class A to class H [4]. SRs recognize various ligands including waste products and invading foreign bodies. These scavenger enzymes usually counteract the production of ROS that is induced by environmental toxins, whereas a dysfunctional host–environment interaction occurs when protective mechanisms are overwhelmed [5]. There are few reports about functional roles of SRs in the maintenance mechanism of homeostasis against oxidative stress in chronic sinus infection.

In the present study, we hypothesized possible roles of SRs in chronic rhinosinusitis (CRS) in relation to underlying pathophysiologic mechanisms that can translate to clinical phenotypes or histological subtypes. For this purpose, we focused on two SRs: lectin-like oxidized LDL receptor-1 (LOX-1) and scavenger receptor class B type 1 (SR-B1). LOX-1 is known to be expressed mainly in the vascular endothelium and inflammatory cells induced by inflammatory cytokines as a result of ischemic changes. The cell surface expression of LOX-1 can be induced by many inflammatory cytokines, oxidative stress, and hemodynamic stimuli [6,7]. SR-B1 was originally known to play a major role in the metabolism of high-density lipoprotein (HDL) with antiatherogenic effects. SR-B1 has been reported as a transmembrane protein that interacts with a broad range of ligands, including lipoproteins, bacteria, and apoptotic cells [8]. A protective function of SR-B1 has also been reported in relation to glucocorticoid production, reduced nitric oxide (NO)-induced cytotoxicity, and increased endotoxin clearance.

The present study is the first to report alterations in LOX-1 expression and production triggered by persistent inflammatory processes in patients with CRS with nasal polyps (CRSwNP). Our findings reveal complex but important roles for SRs that may contribute to the onset of different CRS phenotypes.

## 2. Patients and Methods

### 2.1. Study Design

We conducted a case-control study of 31 patients with CRSwNP and 13 patients with CRS without nasal polyps (CRSsNP), all of whom underwent endoscopic sinus surgery. The diagnosis of sinus disease was based on the patient’s history, clinical symptoms, endoscopic findings, and computed tomography (CT) scanning. Patients with a previous sinus surgery were excluded. None of the patients had received topical or systemic steroids for ≥4 weeks prior to the surgery. The CT images were subjected to radiological grading using the Lund–Mackay system [9]. Nineteen age-matched patients without sinus infection who underwent nose surgery served as controls. They all showed a normal appearance of the paranasal sinus mucosa and normal radiological findings.

### 2.2. RT-PCR Analysis

Mucosal specimens were obtained from the ethmoid sinus and nasal polyps (if any) at the time of surgery. When CRS was present bilaterally, specimens were taken from both sides. The specimens were divided and either snap-frozen or immersed in RNAlater^®^ solution (Ambion, Austin, TX, USA) for RT-PCR or fixed in 4% paraformaldehyde for immunohistochemistry. A quantitative PCR analysis was performed on an ABI Prisms 7300 system (Applied Biosystems, Foster City, CA, USA). Cellular RNA was isolated using RNeasy mini kits (Qiagen, Valencia, CA, USA). Total RNA was then reverse transcribed to cDNA using a High Capacity RNA-to-cDNA kit (Applied Biosystems) according to the manufacturer’s instructions. Gene expression was measured on a real-time PCR system using TaqMan Gene Expression Assays (Life Technologies, Carlsbad, CA, USA). PCR primers specific for LOX-1 (Hs01552593_m1) and SR-B1 (Hs00969821_m1) were used. Primers for GAPDH (Hs03929097_g1) were used as a reference. The PCR cycles were run in triplicate for each sample. Amplifications of the PCR products were quantified by the number of cycles, and the results were analyzed using the comparative cycle threshold (Ct) method (2^−^^ΔΔ^Ct). The quantities of target gene expression are presented as relative rates compared to the expression of the reference gene (ratio: target gene/GAPDH expression).

### 2.3. ELISA Measurement

We measured the serum and sinus tissue concentrations of LOX-1 by using a commercially available ELISA (no. ab212161; Abcam, Cambridge, UK) according to the manufacturer’s instructions. For this purpose, tissue lysates were prepared from fresh frozen sinus specimens of 14 control subjects, 11 CRSsNP patients, and 25 CRSwNP patients. There was no artificial bias for patient selection in the sampling. The obtained specimens were divided into approx. 5-mm^3^ pieces, and the weight of each piece was measured. They were first minced and thoroughly rinsed in phosphate-buffered saline (PBS) to remove blood. Protein extraction from the suspension was performed by a tissue homogenizer using bead beating technology (Precellys^®^ 24, Bertin Technologies, Montigny-le-Bretonneux, France) in 2-mL tubes with 1.4-mm prefilled glass beads (6000 rpm, two cycles for 20 s each). All of the samples were routinely analyzed in duplicate, and the mean values were adopted.

### 2.4. Immunohistochemistry

The primary antibodies used were antihuman SR-B1 rabbit polyclonal antibody (#5193; ProSci, Poway, CA, USA), antihuman LOX-1 rabbit polyclonal antibody (#11837-1-AP; Proteintech, Rosemont, IL, USA), and antihuman CD68 mouse monoclonal antibody (#M0814; Dako, Glostrup, Denmark). Immunostaining was carried out on 5-µm-thick cryostat sections. For antigen retrieval, sections were immersed in Histo VT One (Nacalai Tesque, Kyoto, Japan) at 70 °C for 40 min. The slides were then incubated overnight at 4 °C with the primary antibodies. Color development was achieved using the streptavidin–biotin amplification technique (ChemMate EnVision kit; Dako). Peroxidase activity was visualized by diaminobenzidine solution. Sections were counterstained with hematoxylin. Control specimens developed without the primary antibody were used to verify that nonspecific binding was not detectable. Consecutive sections were stained with hematoxylin and eosin (HE) for the assessment of mucosal pathology and the degree of eosinophil infiltration.

All procedures contributing to this work complied with the ethical standards and with the Helsinki Declaration. The study protocol was approved by the Institutional Review Board at the Hiroshima University School of Medicine on 11 June 2018 (Approval No. Hi-136-2). Written informed consent was obtained from all patients prior to their participation.

### 2.5. Data Analysis

For multiple comparisons, a screening of the data for differences was first carried out using the Kruskal–Wallis test. If the analysis gave a significant result, a further comparison was done by the Mann–Whitney U-test for the between-group analysis. The chi-square test was used to compare qualitative data. Correlation coefficients were calculated by the Spearman method. *p*-values < 0.05 were considered significant.

## 3. Results

### 3.1. Characteristics of Subjects in the Study

The clinical characteristics of the study population are summarized in Table 1. We divided the 44 CRS patients into two groups based on the presence of nasal polyps: the CRSsNP group (n = 13) and the CRSwNP group (n = 31). No significant difference was found among the three groups in the baseline data of age, gender, or body mass index (BMI) distribution. Significant differences were observed between the CRSsNP and CRSwNP groups in the proportion of asthma comorbidity, the severity of computed tomography (CT) scores [9], fractional exhaled nitric oxide (FeNO) levels, and the degree of tissue eosinophils. Further, the CRSwNP patients showed a significantly higher degree of blood eosinophils compared to the control subjects. There was no significant difference in serum LOX-1 levels among the three groups.

### 3.2. Target Genes Expression in Nasal Polyps and Sinus Mucosa

**Figure 1:**
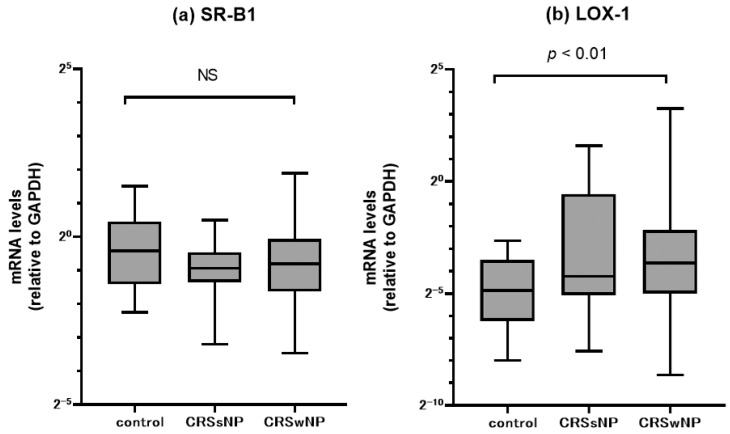
Comparison of mRNA expression in paranasal sinus mucosa from the controls, and CRSsNP and CRSwNP patients as detected by RT-PCR. (**a**) scavenger receptor class B type 1 (SR-B1) and (**b**) lectin-like oxidized LDL receptor-1 (LOX-1) mRNA levels were quantitatively normalized to the glyceraldehyde 3-phosphate dehydrogenase (GAPDH) mRNA levels. Center lines: median values. Boxes: interquartile ranges. Error bars: overall ranges. NS: not significant.

**Figure 2:**
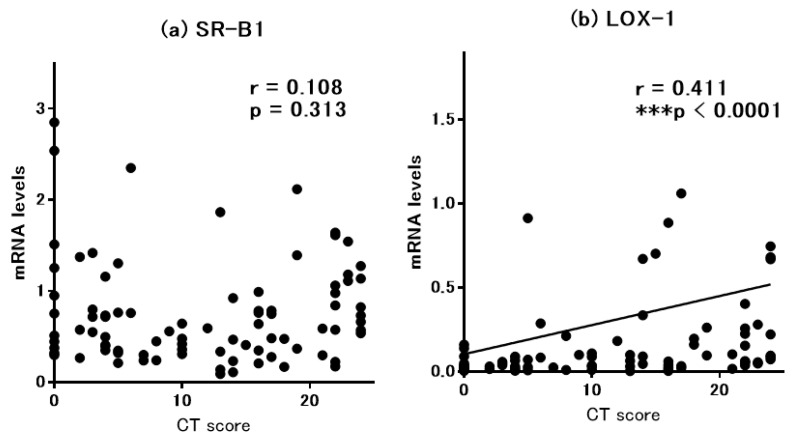
Correlation between the severity of computed tomography (CT) findings and mRNA expression levels for (**a**) SR-B1 and (**b**) LOX-1 in sinus mucosa.

The messenger RNA levels of SR-B1 and LOX-1 in the ethmoid sinus mucosa and nasal polyps were assessed by quantitative RT-PCR (Figure 1). We compared mRNA expression of different SRs in paranasal sinus mucosa between the groups. There was no significant difference in SR-B1 mRNA levels among the three groups. In contrast, the CRSwNP patients showed a significant upregulation of LOX-1 mRNA expression compared to the control subjects. The CRSsNP patients tended to show higher LOX-1 mRNA levels, but the difference versus the controls was not significant. We then examined the relation between the clinical severity of CRS and the gene expression levels. For this purpose, we evaluated the relationship between the mRNA levels of SR-B1 or LOX-1 and the severity of CT scores (Figure 2), and we observed that the LOX-1 but not the SR-B1 mRNA levels were significantly and positively correlated with the CT score (r = 0.411, *p* < 0.0001).

### 3.3. LOX-1 and SR-B1 Proteins Production and Expression

**Figure 3:**
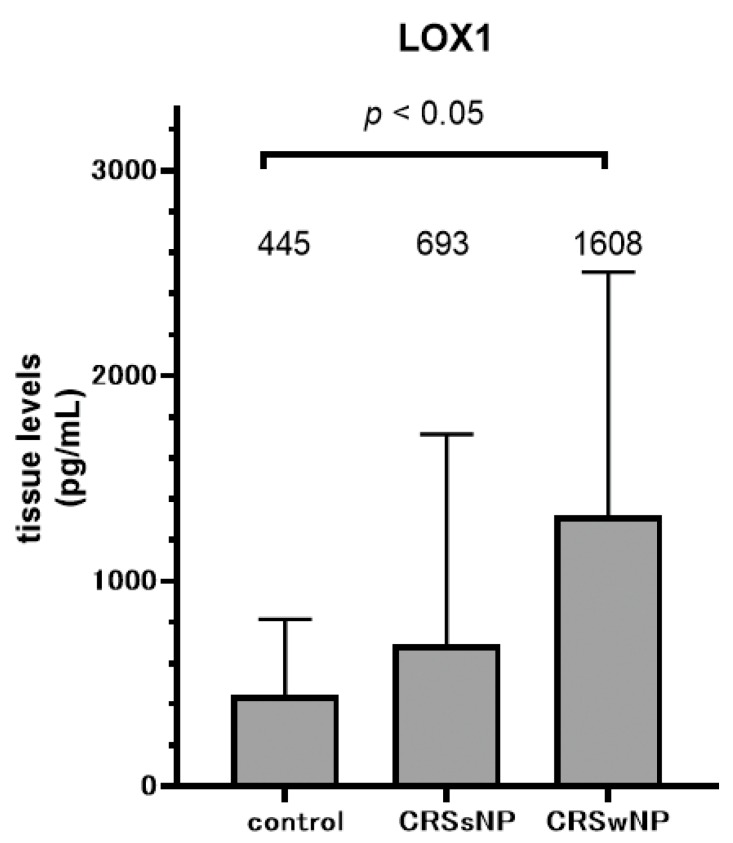
Comparison of tissue levels of LOX-1 protein in paranasal sinus mucosa from controls and CRSsNP and CRSwNP patients as detected by ELISA. Data are mean ± SD.

Since transcriptional changes in LOX-1 were associated with CRS pathology and clinical manifestations, we measured the sinus tissue levels of LOX-1 protein in representative cases (Figure 3). Control subjects showed almost identical LOX-1 protein levels in their serum and tissue samples. On the other hand, the ELISA results of sinus mucosa tissues in the CRS patients revealed elevated concentrations of LOX-1 in contrast to the results in the serum. The mean LOX-1 level in the CRSwNP group was significantly higher than that in the control group.

**Figure 4:**
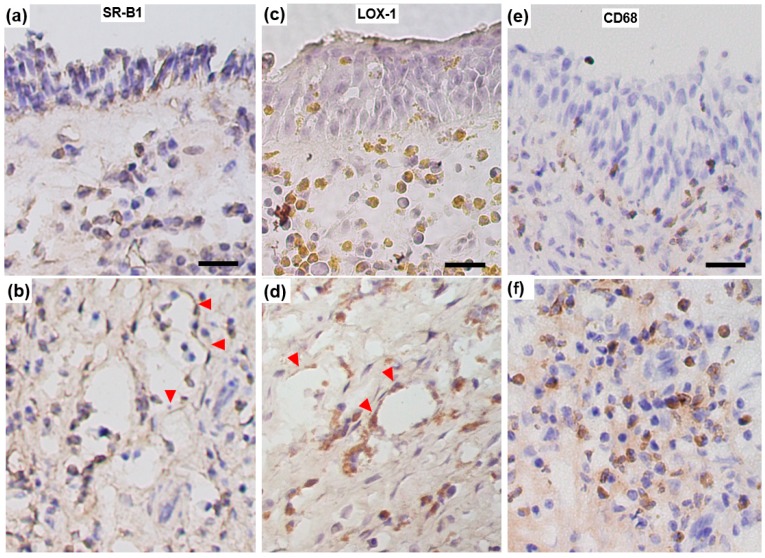
Representative immunohistological images showing SR-B1 (**a**,**b**), LOX-1 (**c**,**d**), and CD68 (**e**,**f**) expression in ethmoid sinus mucosa sampled from a CRSwNP patient. Vascular endothelial cells (arrowheads) are stained positively both for SR-B1 and LOX-1. In contrast, numerous submucosal inflammatory cells show intense positive staining for LOX-1 compared to that for SR-B1. Scale bar: 20 μm.

Figure 4 provides representative immunohistological images of the distributions of SR-B1-, LOX-1-, and CD68-positive cells in the sinus mucosa. In the CRSwNP group, intense inflammatory cell infiltration dominated the ethmoid mucosa and nasal polyps on conventional histological examination. Positive SR-B1 immunoreactivity was localized mainly with associated inflammatory cells and vascular endothelial cells with cytoplasmic staining. The degree of SR-B1 staining appeared to be identical among the three groups. LOX-1 immunoreactivity was also detected in inflammatory cells and vascular endothelial cells with cytoplasmic staining. In contrast, the specimens from the patients in the CRSwNP group generally showed higher rates of intense LOX-1-positive inflammatory cells throughout the submucosal area, with CD68-positive macrophages being predominant.

## 4. Discussion

CRS can be outlined as a dysfunctional host–environment interaction of the paranasal sinus mucosa [10,11,12,13]. It has been proposed that oxidative stress induced by both environmental and intrinsic stimuli underlies the onset and persistency of CRS. The production of ROS has the ability to cause epithelial damage and accentuates mucosal inflammation [14]. However, it has been a matter of debate whether an ischemic condition caused by vascular injury occurs in CRS patients; such a condition is manifested by both bacterial infection and prolonged eosinophilic inflammation [10]. Herein, we investigated the expression and localization of SRs in CRS patients in order to assess the mucosal pathology induced by oxidative stress and ROS production. Our analyses revealed the LOX-1 level was increased in both the mRNA expression and the protein production in the CRSwNP patients. These findings indicate a role of LOX-1 that may contribute to the onset and persistency of chronic paranasal sinus infection based on different CRS phenotypes.

LOX-1 is a major receptor for oxidized low-density lipoprotein (Ox-LDL) produced by oxidative stress [6,7,15]. The binding of ox-LDL to LOX-1 activates the membrane multi-subunit enzyme NADPH oxidase on endothelial cells, resulting in a rapid increase of intracellular ROS. LOX-1 and these complex molecules then modify redox regulation and promote the apoptosis and cell death induced by ROS production. Recent studies have shown that LOX-1 activation is involved in the inflammatory process and inflammatory-related disorders in various organs [16,17].

We identified the distribution of LOX-1 protein in inflammatory cells and vascular endothelial cells in the sinus mucosa. The CRSwNP patients showed higher rates of inflammatory cells that were positive for LOX-1 in the submucosal area where CD68-positive macrophages were densely accumulated. The results were consistent with the quantitative values revealed by ELISA in which the CRSwNP patients exhibited higher LOX-1 levels in the sinus tissue samples compared to those in the control group. A constitutive expression of LOX-1 is observed in vascular endothelial cells in the physiological state. In addition, macrophages and platelets show inducible LOX-1 expression stimulated by inflammatory cytokines as a result of ischemia and vascular injury [18]. The binding of several ligands to LOX-1 induces superoxide generation, inhibits NO production, and enhances the endothelial adhesiveness of leukocytes. These reactions further promote the production of proinflammatory cytokines and elicit the expression of adhesion molecules [19].

In contrast to the increased LOX-1 expression, no significant difference was observed in SR-B1 expression among the present three groups. The immunohistological localization of SR-B1 was mainly confined to the vascular endothelium. SR-B1 belongs to another SR family and plays a major role in the metabolism of HDL cholesterol with antiatherogenic effects [20]. SR-B1 is a multiligand cell surface receptor that mediates the lipid-selective uptake from HDL cholesterol into cells [21]. SR-B1 is also involved in a variety of proinflammatory responses through the interaction with a broad range of ligands [8]. However, it is presumed that the expression of SR-B1 is not affected by CRS pathology.

Another intriguing result in the present study is the positive correlation between the clinical severity of CRS as represented by the CT scores and the LOX-1 mRNA expression level. It has been proposed that the severity and prolongation of CRS are regulated by hypoxia and oxidative stress, leading to angiogenesis through an increase in several growth factors and cytokines. Ischemic conditions in CRS mucosa induce the generation of ROS such as superoxide and hydrogen peroxide, and they cause endothelial cell damage by apoptosis [10].

Our present findings support the theory that a hypoxic environment, in part through a sinus ventilation blockade, drives the LOX-1 expression in inflammatory cells, secondarily triggering an increased production of ROS or peroxynitrite. The downstream signal genes’ expressions and inflammatory response to hypoxic stress by NF-κB activation cause a vicious cycle [22]. Angiogenic factors have also been associated with remodeling of the lamina propria in CRSwNP, suggesting that angiogenesis may be a part of the driving force in polyp formation [23]. Vascular endothelial growth factor (VEGF), a key protein that modulates both angiogenesis and vascular permeability, is more highly expressed in nasal polyp tissue than in CRSsNP or control tissues [24,25]. Our results can therefore be interpreted as a part of the pivotal processes in response to the ischemic condition in the sinus mucosa of CRSwNP patients, which results in a vicious cycle of oxidative stress with inflammatory tissue damage.

Recent reports indicated that serum LOX-1 could also be a potential biomarker for monitoring the severity of inflammation and oxidative stress, or for the prediction of prognoses in patients with arteriosclerosis, obstructive sleep apnea (OSA), or neonatal hypoxic-ischemic encephalopathy [26,27,28]. OSA patients showed higher serum LOX-1 levels compared to controls [28], which might reflect a critical role of systemic inflammation in the development and progression of OSA. In the present study, we found no significant difference in serum LOX-1 levels, in contrast to the tissue levels. This finding, together with the lack of differences in the BMI values and underlying disease among the three groups, leads us to speculate that the accumulation of LOX-1 in the sinus mucosa is unlikely to affect the systemic hemodynamics.

Another topic that deserves further attention is the possible involvement of NO and its oxidative metabolites, referred to as reactive nitrogen species (RNS). The human paranasal sinuses produce physiologically large amounts of NO and play a role as a physiological reservoir [29]. Increased NO production causes the production of peroxynitrite from superoxide and NO in inflammatory cells, leading to severe cell damage by lipid peroxidation and tyrosine nitration in the airway epithelium of asthmatic patients [30,31]. In the present study, 16 of the 31 patients in the CRSwNP group had a history of bronchial asthma, and the mean FeNO level in this group was higher than that in the CRSsNP group, which is consistent with previous reports [32]. We can therefore propose that augmented LOX-1 expression in the CRSwNP patients induces an increased production of ROS and promotes crosstalk with NO.

There are some study limitations. It was a cross-sectional study, and the data collections excluded the patients’ postoperative courses. An established method to assess ischemic conditions in the paranasal sinuses has not been available for clinical use. In summary, our findings reveal novel and important roles for LOX-1 in the local redox regulation that may contribute to the pathogenesis of CRS. Further research may elucidate the NO homeostasis and oxidant stress in the upper airways as well as therapeutic interventions by antioxidant agents.

## Figures and Tables

**Table 1:** Demographics and clinical background of the study population.

	Control Group (n = 19)	CRSsNP Group (n = 13)	CRSwNP Group (n = 31)
Males/Females	9/10	8/5	14/17
Age	46.4 (19.4)	50 (18.6)	56.2 (13.2)
Allergic rhinitis	10 (52.6%)	10 (76.9%)	20 (64.5%)
BMI, kg/mm^2^	22 (2.5)	22.5 (2.8)	22.6 (3.3)
Bronchial asthma	1 (5.2%)	3 (23%)	14 (45.1%) **
Blood eosinophils, %	2.9 (2.5)	4.1 (2.9)	6.4 (4.0) ^††^
FeNO, ppb	21.33 (19.16)	16.54 (12.65)	32.13 (27.86) ^†^
Tissue eosinophils, cells/HPF	15.6 (26.2)	30.1 (44.8)	133.8 (111.3) ***
CT score	-	6.5 (4.3)	15.9 (6.5) ***
LOX-1 serum levels, pg/mL	417 (264.7)	473.8 (329.5)	585.7 (390.2)

Data are the mean ± standard deviation (SD) or the number with percentages in parentheses. ** *p* < 0.01, *** *p* < 0.001 vs. the other groups. † *p* < 0.05 vs. the CRSsNP group. †† *p* < 0.01 vs. the control group. BMI: body-mass index, CRSsNP: chronic rhinosinusitis without nasal polyps, CRSwNP: chronic rhinosinusitis with nasal polyps, FeNO: fractional exhaled nitric oxide, HPF: high power field (x400).

## Data Availability

The datasets used and analyzed in the study are available from the authors on reasonable request.
